# Preparation and Application of Starch/Polyvinyl Alcohol/Citric Acid Ternary Blend Antimicrobial Functional Food Packaging Films

**DOI:** 10.3390/polym9030102

**Published:** 2017-03-14

**Authors:** Zhijun Wu, Jingjing Wu, Tingting Peng, Yutong Li, Derong Lin, Baoshan Xing, Chunxiao Li, Yuqiu Yang, Li Yang, Lihua Zhang, Rongchao Ma, Weixiong Wu, Xiaorong Lv, Jianwu Dai, Guoquan Han

**Affiliations:** 1 School of Mechanical and Electrical Engineering, Sichuan Agricultural University, Ya’an 625014, China; wzj@sicau.edu.cn (Z.W.); 13908160316@163.com (L.Z.); 13608265802@163.com (R.M.); 13350568176@163.com (W.W.); 2 School of Food Science, Sichuan Agricultural University, Ya’an 625014, China; 13350966938@126.com (J.W.); 18227590905@126.com (T.P.); 18227553959@126.com (Y.L.); 18227592524@126.com (C.L.); 13822267735@126.com (Y.Y.); 18227591911@126.com (L.Y.); 13795853723@163.com (X.L.); 18728187200@163.com (J.D.); dahan980306@126.com (G.H.); 3 Stockbridge School of Agriculture, University of Massachusetts, Amherst, MA 01003, USA

**Keywords:** packaging films, antimicrobial, figs (*Ficus carica* L.)

## Abstract

Ternary blend films were prepared with different ratios of starch/polyvinyl alcohol (PVA)/citric acid. The films were characterized by field emission scanning electron microscopy (FE-SEM), thermogravimetric analysis, as well as Fourier transform infrared (FTIR) analysis. The influence of different ratios of starch/polyvinyl alcohol (PVA)/citric acid and different drying times on the performance properties, transparency, tensile strength (TS), water vapor permeability (WVP), water solubility (WS), color difference (ΔE), and antimicrobial activity of the ternary blends films were investigated. The starch/polyvinyl alcohol/citric acid (S/P/C^1:1:0^, S/P/C^3:1:0.08^, and S/P/C^3:3:0.08^) films were all highly transparent. The S/P/C^3:3:0.08^ had a 54.31 times water-holding capacity of its own weight and its mechanical tensile strength was 46.45 MPa. In addition, its surface had good uniformity and compactness. The S/P/C^3:1:0.08^ and S/P/C^3:3:0.08^ showed strong antimicrobial activity to *Listeria monocytogenes* and *Escherichia coli*, which were the food-borne pathogenic bacteria used. The freshness test results of fresh figs showed that all of the blends prevented the formation of condensed water on the surface of the film, and the S/P/C^3:1:0.08^ and S/P/C^3:3:0.08^ prevented the deterioration of figs during storage. The films can be used as an active food packaging system due to their strong antibacterial effect.

## 1. Introduction

Food packaging is an important part of food products, both to protect food quality and safety of food products to enhance their added value. Food packaging materials with sufficient mechanical strength, barrier properties, thermal stability, biodegradability, and antibacterial and antioxidant properties are necessary for food safety and extending the shelf life of packaged foods. Currently, the materials used in packaging industries are dominated by petroleum based plastic materials produced from fossil fuels since they are relatively cheap and convenient to use with good process capability and durability [1]. However, due to the non-biodegradable nature of petroleum-based plastics, the environmental pollution caused by traditional plastic packaging is becoming more and more serious, and thus there is a need to develop new packaging materials. With the development of modern biotechnology, biodegradable films as environmentally friendly materials are being paid increasing attention, and have become a new generation of hot research and development projects as well as becoming a part of the basic strategy included in a global economic sustainable development [2]. Natural polymer materials such as starch, which are biodegradable products, are renewable resources with low cost and great potential advantages. However, their poor barrier properties, mechanical and processability, compared to petroleum-based plastic materials, are a major limitation in the use of biopolymer films for food packaging applications. Generally, natural polymers are used to blend with nanomaterials or other synthetic polymers with the aim of extending their applications [1]. Currently, the mechanical, barrier, and antibacterial properties of the composite films compounded of nanomaterials and other materials such as silver, ZnO, TiO_2_, are being studied by a variety of laboratory techniques and film coating methods. However, nanomaterials have not been widely used in industry. Poly (vinyl alcohol) (PVA) is a biodegradable synthetic polymer, which is a kind of thin film material with excellent performance and wide application. The combination of PVA and starch improved the degradation of starch-filled biodegradable plastic [3]. In addition, the research results on the composite film of polyvinyl alcohol (PVA) with a variety of materials, including essential oils, modified nano-materials etc., proved its good packaging performance, and the existence of film pores and the size of the loading affect the amount of antimicrobial agents, thus affecting the antibacterial properties of the film [4,5,6,7,8,9]. The use of biopolymers as substitutes for non-degradable traditional plastics is an interesting alternative still for short-term applications. In order to improve the fresh-keeping performance, the antibacterial property of the films is very necessary. Until now, the research status of antibacterial biodegradable cling films was as follows: As early as 1997, Zhao et al. found that TiO_2_ had a photocatalytic capacity, for the micro-organisms and toxins produced by decomposition. After that, photocatalytic antimicrobial agents began to develop rapidly [10]. At present, environmentally friendly films that incorporate organic contaminants into ordered mesoporous materials have been used in food packaging [11,12,13,14,15]. In addition, through the analysis of ginger oil, thyme oil, grapefruit, peach leaf extract and so on, it was found that the essential oil had antibacterial effect, and proved that the prepared plastic wrap could save the food to achieve the effect of preservation and antibacterial [14,16,17,18].

Recently, there have been many studies on figs, the subjects of this experiment, such as the life cycle and nutritive value of figs, the changes of physiological and storage quality, the effects of different storage temperature, the effects of different packaging materials and the effect of 1-MCP treatment on the storage quality of figs [19,20,21,22,23]. The results showed that the figs have high nutritional value, good antibacterial, antifungal and anti-cancer activities as well as others [24,25]. In previous work, citric acid was added to the film, the different characteristics of film preservation were analyzed, and their antibacterial and biodegradability were discussed [26,27]. However, due to the few reports that have been made on films with high mechanical and thermal properties, the preparation of PVA/starch based biodegradable antibacterial films has practical significance and is of great significance in the field of food packaging. Therefore, the purpose of the present study is to use starch and polyvinyl alcohol composite, modified to obtain films with better antibacterial, mechanical, and thermal properties, to prepare PVA/starch based citric acid biodegradable antimicrobial films.

## 2. Materials and Methods

### 2.1. Materials

Citric acid, (Tianjin Bodi Chemical Co., Ltd., Tianjin, China); corn starch, food grade, (Zhuhai Jindu Tide Food Co., Ltd., Zhuhai, China); glycerol, analytical pure, (Wuxi Yatai United Chemical Co., Ltd., Wuxi, China); polyvinyl alcohol (Shanghai Yingjia Industrial Chemical Co., Ltd., Shanghai, China); distilled water, (laboratory homemade).

DZKW-D-1 electric heating thermostatic water bath (Zhengzhou North and South Instrument Co., Ltd., Henan, China); FA2204B analytical balance (Guangzhou Ruiming Instrument Co., Ltd., Guangzhou, China); TWCL-T electronic thermostat (Shanghai Branch equipment Co., Ltd., Shanghai, China); JJ YZG/FZG vacuum dryer (Changzhou Nile Drying Equipment Co., Ltd., Changzhou, Jiangsu, China); WDW-20 computer controlled electronic universal testing machine (Beijing Dana Machinery Co., Ltd., Beijing, China); CH-1-B hand count (Shanghai music magnetic instrument company, Shanghai, China); Thermogravimetric analyzer (Leco TGA 701; Leco, St Joseph, MI, USA); FE-SEM (LINE No. 337, 338, S-4800) [Hitachi Co., Ltd., Matsuda, Japan]; ATR FT-IR, (Billerica, MA, USA).

### 2.2. Preparation of Films

Starch/Polyvinyl alcohol (S/P) ternary blend functional food packaging films were prepared by using the solvent casting method [28]. Film solution were prepared by dissolving 2.81 g of starch, polyvinyl alcohol into 30 mL of distilled water with 2.11 g of glycerol as a plasticizer while mixing vigorously for about 45 min at 95 °C using an electric stirrer. The S/P and citric acid composite films (S/P/C) were prepared by the solution casting method as described by Wang et al. [28]. First, PVA was dissolved in distilled water at 95 °C while the corn starch was gelatinized at 90 °C. Thereafter, citric acid was added to the PVA solution at 80 °C, gelatinized starch and glycerin were added with stirring for 30 min. A transparent and uniform film fluid was obtained.

All the film solutions were cast onto leveled glass plate (25 cm × 25 cm) and were dried for about 24 h at room temperature and peeled off from the plate to obtain a dried film. The film thickness was measured using a micrometer (40SH/SD, Mahr, Goettigen, Germany) with an accuracy of 0.01 mm. All films samples were preconditioned in a constant temperature humidity chamber set at 25% and 50% RH for at least 48 h before further testing (Table 1).

### 2.3. Surface Color and Transparency of Films

The surface color of the films was measured using a white color plate (*L* = 97.75, *a* = −0.49, and *b* = 1.96) as a standard background for color measurement [29]. Total color difference (DE) was calculated as follows:

(1) $$\Delta E={\left[{\left(\Delta L\right)}^{2}+{\left(\Delta a\right)}^{2}+{\left(\Delta b\right)}^{2}\right]}^{0.5}$$

Δ*L* means bright and dark, + is bright, − indicates darkness; Δ*a* for red and green, + for reddish, and for partial green; Δ*b *represents yellow and blue, + indicates yellowish, − indicates blue.

### 2.4. Surface Morphology and FTIR Analysis

The films were cut to a size of 1 mm × 4 mm. The surfaces of the films were fixed with conductive adhesive and were sprayed with gold. The composite films were observed using a field FE-emission scanning electron microscopy (FE-SEM (LINE No. 337, 338), S-4800, Hitachi Co., Ltd., Matsuda, Japan) operated at an acceleration voltage of 1 kV [29]. By using Spectrum 100 Fourier transform infrared spectroscopy (FT-IR), spectroscopy analysis was performed and the three kinds of films tested with a KBr tablet [30]. The analysis conditions of the different starch composite film samples ATR-FTIR all had a scanning frequency of 128 with spectral resolution of 4 cm ~ (−1) where the wave-number ranged from 4000 to 400 cm^–1^.

### 2.5. Mechanical Properties

The mechanical properties of the films were analyzed by measuring the tensile strength (TS) and elongation at break (*E*) according to the standard ASTM method D 882-88 using an Instron Universal Testing Machine (Model MDX, Instron Engineering Corporation, Canton, MA, USA) equipped with a 0.5 kN load cell. Each film was cut into rectangular strips (3 cm × 8 cm). The machine was operated in tensile mode with an initial grip separation of 50 mm and crosshead speed of 50 mm/min. The TS was determined by dividing the maximum load (*N*) by the initial cross-sectional area (m^2^) of the films and expressed in MPa. The *E* (%) was determined by dividing the extension at rupture of the films by the initial length of the films (50 mm) multiplied by 100 [28].

Each sample was tested three times and averaged. Then the TS of the films was calculated using the following equation:

(2) $$T_{S}=\frac{F}{S}$$

In the formula:

*Ts*—Tensile strength, MPa;
*F*—The maximum tensile force when the sample breaks, N;
*S*—Cross-sectional area of specimen, m^2^.

### 2.6. Water Vapor Permeability (WVP)

The water vapor transmission rate (WVP) of films was determined gravimetrically at 25 °C under 50% RH conditions using water vapor transmission measuring cups in accordance with the ASTM E96-95 standard method. Each sample was measured five times [31]. Then, the WVP of the films was calculated using the following Equation (3):

(3) $$\mathrm{WVP}=\frac{WVTR\times n\times K}{△p}$$

In the formula:

WVP—Water vapor transmission coefficient, ×10^–9^ g·m/(m^2^·Pa·s);
*WVTR*—The amount of water vapor transmitted through the instrument was measured, g/(m·d);
*n*—Film thickness, mm;
Δ*p*—The output pressure of the gas is 0.20 MPa.

### 2.7. Determination of Solubility of Cling Film References

When the dissolution rate was measured, the films (20 mm × 20 mm) were dried in a constant temperature blast oven at 70 °C for 24 h and taken as the initial weight of the films [28]. The films were then placed in 100 mL of deionized water and were taken out after 24 h to dry off surface moisture. The remaining films were placed in a constant temperature blast oven at 70 °C for 24 h to obtain the weight of the final films, mt. The dissolution rate is calculated according to the equation:

(4) $$D=\frac{\left(m_{0}-m_{t}\right)}{m_{0}}\times 100\%$$

In the formula:

*D*—Dissolution rate, %;
*M*_0_—Initial film weight, g.

### 2.8. Thermal Stability

The thermal stability of film samples was evaluated using a thermogravimetric analyzer (Leco TGA701; Leco, St Joseph, MI, USA). About 25 mg of film sample was taken in a standard aluminum cup and heated from 25 to 600 °C with a heating rate of 12 °C/min under a nitrogen flow (50 cm^3^/min). An empty cup was taken as a reference. The derivative form of TGA (DTG) was obtained by calculating the differentials of the TGA values using a central finite difference method as follows:

(5) $$\mathrm{DTG}=\frac{\left(W_{t+\Delta t}-W_{t+\Delta t}\right)}{2\Delta t}$$

where *W_t_*_+Δ*t*_ − *W_t_*_−Δ*t*_ are the residual weight of sample at time *t* + Δ*t* and *t* − Δ*t*, respectively, and Δ*t* is the time interval for reading the residual sample weight [32].

### 2.9. Antibacterial Activity

The antibacterial activities of S/P/C^1:1:0^, S/P/C^3:1:0.08^, and S/P/C^3:3:0.08^ films were examined for their inhibitory effects against the growth of Gram-positive bacteria, *L. monocytogenes*, and Gram-negative bacteria, *E. coli*. *L. monocytogenes*. *E. coli* were aseptically inoculated in 20 mL BHI (brain infusion) and TSB (trypsin soy broth) broth, respectively and subsequently incubated at 37 °C for 15 h. Each cultured broth was centrifuged at 4000 rpm for 10 min and the cell pellets were suspended in 100 mL of sterile TSB and BHI broth respectively, and diluted 10 times with sterile distilled water. Then 50 mL of diluted broth (10^6^e10^7^ CFU/mL) was taken into 100 mL of the conical flask containing films sample (5 cm × 5 cm) and subsequently incubated at 37 °C for 12 h under mild shaking. The same diluted broth without film sample was used as the control. At every 3 h interval, the cell viability of each pathogen was calculated by absorbance value which was determined at 600 nm with a spectrophotometer. Antimicrobial tests were performed in triplicate with individually prepared films [28].

### 2.10. Packaging Test

The figs were wrapped with plastic wrap and the physiological indexes were measured.

#### 2.10.1. Determination of Malondialdehyde (MDA) Content

Take 1 g of the fig in a bowl with 10 mL of Tris-HCl buffer (0.1 mol·L^–1^ pH 8.5) added and then mix well and grind into a homogenate. All the homogenate is transferred into a centrifuge tube and centrifuged at 4000 rpm (4 °C) for 5 min. An amount of 1.5 mL of the supernatant is used（The control group is added with 1.5 mL of 10% TCA solution) with 2.5 mL of 0.5% TBA solution added, mixed up and then reacted in boiling water for 15 min, rapidly cooled down and then centrifuged (if clear supernatant no need to centrifuge). The supernatant was measured for absorbance at wavelengths of 450, 532, and 600 nm with a spectrophotometer.

(6) $$MDA(\mu \mathrm{mol}\cdot \mathrm{FW}\cdot g^{-1})=\left[6.45\times (OD_{532}-OD_{600})-0.56\times OD_{450}\right]\times V\times \frac{V_{1}}{V_{2}}\times M$$

*V*—Volume of extract, mL;
*V*_1_—Volume of reaction, mL;
*V*_2_—Determined volume of extract, mL;
*M*—Fresh weight of plant tissue, g.

#### 2.10.2. Determination of Ascorbic Acid Content

Grind the fig into a homogenate, take 15 g in a 100 mL volumetric flask, add 1% oxalic acid to the scale line and then filter with absorbent cotton. Take 10 mL of the filtrate, place it into a 100 mL beaker, and add 1 mL of 1% starch and 20 mL of 1% oxalic acid. Mix them and titrate to blue with standard iodine keeping 15 s without fading, write down the date. Do three parallel experiments and take the average, also do a blank test.

(7) $$X=\frac{H\times (V_{1}-V_{2})}{M}\times 100$$

*X*—mg of ascorbic acid per 100 g of figs, mg/(100 g);
*H*—Concentration of standard iodine, mg/mL;
*V*_1_—Consumption of standard iodine titration volume, mL;
*V*_2_—Consumption of standard iodine solution volume by blank titration, mL;
*M*—Quality of the sample, g.

#### 2.10.3. Determination of Reducing Sugar Content

Take 10 g homogenate of the fig, transfer into a 250 mL volumetric flask, slowly add 5 mL of zinc acetate solution and 5 mL of potassium ferrocyanide solution, dilute with water to the mark, shake it and let it stand for 30 min. Filter with a dry filter, discard the early filtrate and collect the filtrate in a 250 mL conical flask in reserve.

Accurately draw 5 mL each of the basic copper tartrate solution A and B into a 100 mL conical flask, add 10 mL of distilled water and 3 pieces of glass beads. Then add 9 mL standard glucose solution (1 mol/L) into the conical flask and heat to boil in 2 min. Add a standard glucose solution until the blue solution just fades. Do three parallel experiments to obtain the average.

(8) F=C×V

*F*－10 mL of basic copper tartrate solution corresponds to the mass of glucose, mg;
*C*－Concentration of standard glucose solution, mg/mL;
*V*－The volume of the standard glucose solution consumed during calibration, mL.

Determination of the sample solution: Take 5 mL each of the basic copper tartrate solution A and B, place them in a 100 mL conical flask and then add 10 mL distilled water and 3 pieces of glass beads. Add the sample to the burette and add to the boiling solution until the blue solution just fades at the end. Carry out three times to obtain the average.

(9) $$X=\frac{F}{M\times \frac{V}{250}\times 1000}\times 100$$

*X*—mg of reducing sugar per 100 g of figs, mg/(100 g);
*M*—Weight of sample, g; F-10 mL of basic copper tartrate solution corresponds to the mass of glucose, mg;
*V*—The volume of the sample solution consumed in the assay, mL;
250—Total volume of sample solution, mL.

#### 2.10.4. Determination of Titratable Acid Content

Take 20 g of homogenate (accurate to 0.001 g), place in 250 mL volumetric flask, dilute with water to the mark. Hold it for 30 min and shake 2 or 3 times during this time. Filter with absorbent cotton and collect the filtrate in a 250 mL conical flask in reserve.

Take 20 mL of the filtrate in the conical flask, add 2 drops of phenolphthalein indicator, titrate to pink color with calibrated NaOH solution (0.011 mol/L) for 30 s without fading and record the amount of NaOH solution. Do three parallel experiments for every sample to obtain the average, and carry out a blank test.

(10) $$X=\frac{C\times (V_{1}-V_{2})\times K}{M}\times 100$$

*X*—Number of grams of acid per 100 g of figs, g/(100 g);
*C*—Concentration of sodium hydroxide standard titration solution, mol/L;
*V*_1_—The volume of the standard sodium hydroxide solution consumed, mL;
*V*_2_—The volume of the standard sodium hydroxide solution consumed in the blank experiment, in mL;
*K*—Conversion factor of acid, 0.067 in malic acid;
*M*—Weight of sample, g.

#### 2.10.5. Determination of Polyphenols

There are many methods for the determination of polyphenols, but we decided to use high performance liquid chromatography (HPLC) in consideration of the experimental devices and other factors. In addition, it was necessary to prepare ferrous tartrate solution: Weigh 1 g of ferrous sulfate and 5 g of potassium sodium tartrate, dissolve in water and make up to 1 L (the liquid was stored overnight before use and can be stable for 1 week).

Accurately weigh 1 g of grated figs in a 250 mL beaker, add 80 mL of boiling water, hold in boiling water for 30 min and then filter the liquid in the beaker, wash, transfer the filtrate into a 100 mL volumetric flask. The liquid is cooled to room temperature and finally diluted to the scale line with distilled water, and shaken evenly. Take 1 mL of the sample solution into a 25 mL volumetric flask, add 4 mL of distilled water, 5 mL of ferrous tartrate solution in order, shake, and then add the phosphate buffer (pH = 7.5) to the scale line. The sample solution is replaced without ferrous tartrate solution as a blank experiment. The absorbance values are determined at a wavelength of 540 nm with a colorimetric cup of 5 cm.

(11) $$P=A\times \frac{7.826}{1000}\times \frac{V_{1}}{V_{2}\times m}\times 100\%$$

*P*—Content of polyphenols, g/100 mL;
*A*—Absorbance of sample solution;
*V*_1_—Total sample solution, mL;
*V*_2_—The amount of test solution taken, mL;
*M*—Quality of sample, g.

#### 2.10.6. Determination of the Activity of Catalase (CAT)

Take one gram of figs (Chengdu, China) for pre-cooling, add 20 mL of phosphate buffer (pH = 7.8), grind into slurry in an ice bath, transfer it into a 25 mL volumetric flask and then flush the portland with the buffer. Add the phosphate buffer to the scale line and put the volumetric flask in the fridge at 5 °C.

Let it stand for 10 min. Next, put it into a centrifugal tube and centrifuge at 4000 r/min (4 °C) for 15 min. Preserve the supernatant at the low temperature. The reaction system consists of 2.9 mL, 20 mol/L of H_2_O_2_ and 0.1 mL of the supernatant with distilled water as blank control. Begin to record after 15 s from the start of the reaction. Absorbance is measured at a wavelength of 240 nm and the data taken as the initial data. Record a data point every 30 s and it is necessary to measure continuously to obtain six data. Carry out three parallel experiments. Take the reducing absorbance of 0.01 per gram of sample per minute as a unit of the catalase’s activity. The unit is 0.01 ΔOD 240 min^–1^·g^–1^ fresh weight (FW).

### 2.11. Statistical Analysis

Films properties were measured with individually prepared films in triplicate, as the replicated experimental units and the results were provided with mean ± SD (standard deviation) values. One-way analysis of variance (ANOVA) was performed, and the significance of each mean property value was determined (*p* < 0.05) with the Duncan’s multiple range test of the statistical analysis system using the SPSS computer program (SPSS, Inc., Chicago, IL, USA).

## 3. Results and Discussion

### 3.1. Apparent Color and Optical Properties of Films

All the film solutions for the preparation of S/P/C^1:1:0^, S/P/C^3:1:0.08^, and S/P/C^3:3:0.08^ formed uniform and standing films. Apparently, the S/P/C^3:3:0.08^ was clear and transparent with high lightness (high Hunter L-value of 71.2) as shown in Table 2. The S/P/C^3:1:0.08^ and S/P/C^3:3:0.08^ composite films maintained high transparency with slight decrease in lightness and the slight increase in yellowness as shown in the decreased Hunter-b values, respectively. The increase in yellow tint of the S/P/C^3:1:0.08^ and S/P/C^3:3:0.08^ composite films was mainly attributed to the polyphenols compounds included in the citric acid [33].

### 3.2. Microstructure and Fourier Transform Infrared (FTIR) Analysis

**Figure 1:**
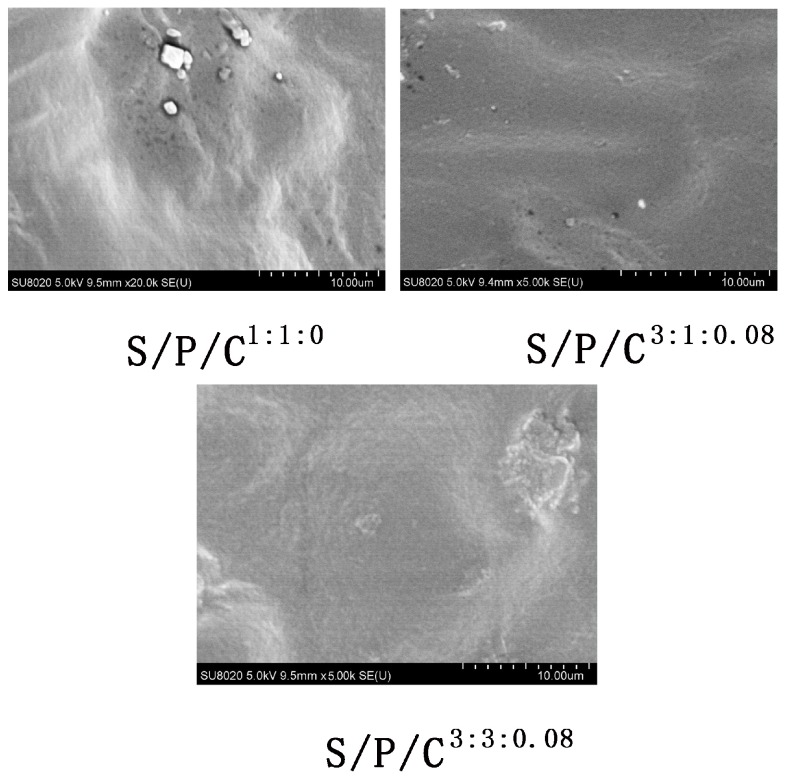
FE-SEM images of starch/polyvinyl alcohol/citric acid ternary blend functional food packaging films.

Microstructure of the films was evaluated using FE-SEM and the resulting FE-SEM images of surface morphology of the films are shown in Figure 1. The FE-SEM images of the films showed that all the films had a uniform and smooth surface. As can be seen from the surface topography of the films, the surfaces are homogeneous, smooth, and continuous, no pores appear, and the surfaces of the films are continuous and dense. The white granular material may be starch granules, and the starch granules will reduce the mechanical properties. S/P/C^3:1:0.08^ and S/P/C^3:3:0.08^ showed that the surfaces of the composite films were free of projections and wrinkles, and the phase separation interface between PVA and starch was not significant. This indicates that glycerol and citric acid can significantly improve the binding of starch and polyvinyl alcohol and enhance the dense homogeneity of the film. FE-SEM images of the control and cross-linked films do not show any appreciable change in surface morphology due to cross-linking, as seen from Figure 1. The S/P/C^3:1:0.08^ and S/P/C^3:3:0.08^ were homogenous without pores or cracks and the starch molecules had been well dispersed without the many granules that were observed in films made from starch mixed with PVA.

**Figure 2:**
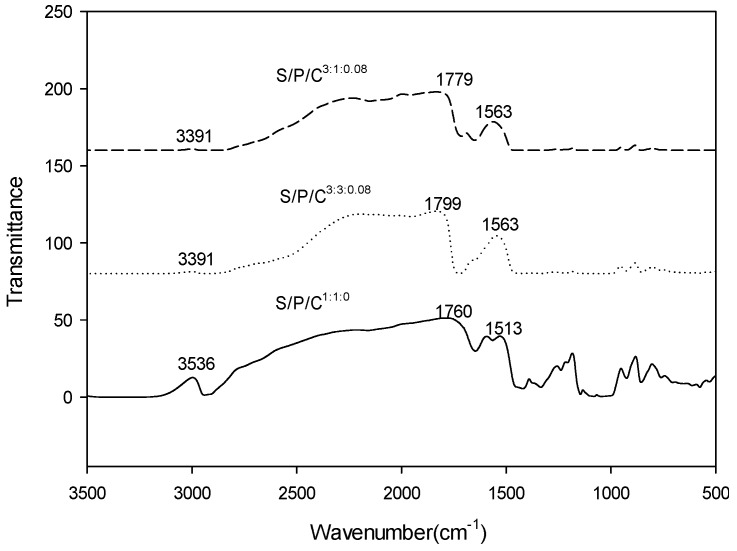
FTIR spectra of starch/polyvinyl alcohol/citric acid ternary blend functional food packaging films.

FTIR analysis of the films was carried out to study the interactions between fillers and polymer matrix and the resulting FTIR spectra are shown in Figure 2. The absorption peak observed at 3391 cm^−1^ is related to the stretching vibration of O-H in the starch and PVA structures [33,34,35]. The peaks at 1779 cm^–1^ correspond to the carboxyl and ester carbonyl bands [36]. The spectra of S/P/C^3:3:0.08^ and S/P/C^3:1:0.08^ films shows that the peak band increases and the peak intensity increased compared with the S/P/C^1:1:0^ film. The intensity of the band increases and the values of the peak band migrate from 3391 to 3536 cm^–1^, from 1779 to 1760 cm^–1^, and from 1563 to 1513 cm^–1^. The results show that the characteristic absorption peaks are consistent with the relative published results [32]. These changes may be related to starch, glycerol, PVA content of different ratios, and the addition of citric acid modifies PVA, resulting in modified PVA with starch association enhanced.

### 3.3. Mechanical Properties

Mechanical properties such as tensile strength (TS), elongation at break (E), of the S/P/C^1:1:0^ blend films are shown in Table 3. Thickness of the S/P/C^3:1:0^ and S/P/C^3:3:0.08^ blend films increased slightly by the addition of citric acid, which is mainly due to the increased solid content. The TS, which indicates the strength of film, of the S/P/C^1:1:0^ control films was 35.98 ± 1.8 MPa. Although the strength of the S/P/C^3:3:0.08^ films was lower than that of the agar/carrageenan/konjac ternary blend film prepared with a similar method as the present study [37], it was comparable to those of commodity plastic films such as high density polyethylene (22–23 MPa), low density polyethylene (19–44 MPa), and polypropylene (31–38 MPa) [38]. The optimum baking time for each of the three films was 270 min. However, as time increased to 300 minutes, a long bake caused the films to crack, resulting in a sharp drop in TS. Yet, compared with the S/P/C^1:1:0^ films, the TS of the composite films with citric acid added was significantly increased (*p* < 0.05), including the S/P/C^3:3:0.08^ and the S/P/C^3:1:0.08^. The increase of mechanical strength is mainly due to the physical attraction between the polymer matrix PVA and citric acid, and the polycarboxylation of citric acid with the alcoholic hydroxyl groups of PVA, as shown by FTIR results. The distribution of citric acid with high elastic modulus generate tremendous interfacial contacts with the polymer matrices, which leads to effective stress transfer resulting in an increase in the TS [37]. On the contrary, the flexibility of S/P/C^3:3:0.08^ composite films decreased slightly while that of S/P/C^3:1:0.08^ composite films increased slightly compared with the control S/P/C^1:1:0^ blend film, as indicated by the E values. The E of a film is usually inversely proportional to the TS of the films as shown in the present study. The slight increase of flexibility (i.e., increase in E) of the S/P/C^3:1:0.08^ composite films can be attributed to the higher amount of glycerol (a plasticizer) accompanied by the citric acid. Glycerol acts as a plasticizer without forming any covalent linkages with the biopolymer. The hydroxyl groups present in glycerol are expected to form hydrogen bonds with the biopolymer molecules at the carbonyl and hydroxyl sites. Being small in size, this effectively increases the free volume of the system, thus decreasing the glass transition temperature and intermolecular forces. As a result, the plasticized biopolymer matrix changes from brittle to leathery to rubber with increased flexibility and extensibility of the film. Manufacturer's information indicated that the citric acid contained 30% of glycerol.

### 3.4. Water Vapor Permeability (WVP)

The WVP of the S/P/C^1:1:0^ was determined by a gravimetric method using WVP cups and the results are shown in Table 4. The WVP value of the control films was (1.56 ± 0.09) × 10^–9^ g m/m^2^·Pa·s which is comparable to the usual carbohydrate biopolymer films [37]. While the WVP of S/P/C^3:1:0.08^ composite films was not significantly different from that of the control S/P/C^1:1:0^ blend films, that of S/P/C^3:3:0.08^ composite film decreased significantly (*p* < 0.05) compared with the control film. Such decrease in the WVP has been frequently observed with other biopolymers composited with citric acid [37,39]. The starch nanocrystals obtained by removing the amorphous parts of the original starch granules by acid hydrolysis under the gelatinization temperature are compact, have high rigidity, high crystallinity, and low moisture permeability due to their disc shape. When starch paste was added dropwise to PVA, the starch reassembled with polymerization to form nanoprecipitation by intermolecular or intramolecular hydrogen bond interaction. This is probably due to the fact that the starch nanocrystals are susceptible to forming a well intercalated nanocomposite structure and form nano-precipitates with the modified organic citric acid which acts as a reinforcing agent to modify the polyvinyl alcohol. Thus, the permeability of the water vapor due to the permeability of the starch/PVA nanoparticles leads to twists and turns [28,35]. The water solubility of S/P/C^1:1:0^ was high, because its main components were starch and polyvinyl alcohol cross-linked polymer. The results are shown in Table 4. The addition of citric acid resulted in the films cross-linking more closely, and the S/P/C with the same ratio of starch and polyvinyl alcohol had better water solubility. On the contrary, the water soluble effect of S/P/C^3:3:0.08^ was worse than that of S/P/C^3:1:0.08^, because it had a large amount of PVA, which did not have the cross-linking effect. Citric acid can thus enhance the water solubility of the film.

### 3.5. Thermal Stability

**Figure 3:**
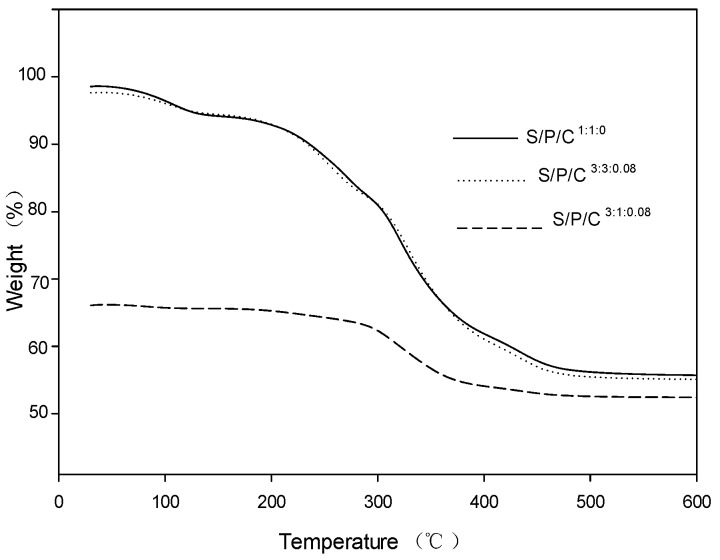
TGA thermograms of starch/polyvinyl alcohol/citric acid ternary blend functional food packaging films.

**Figure 4:**
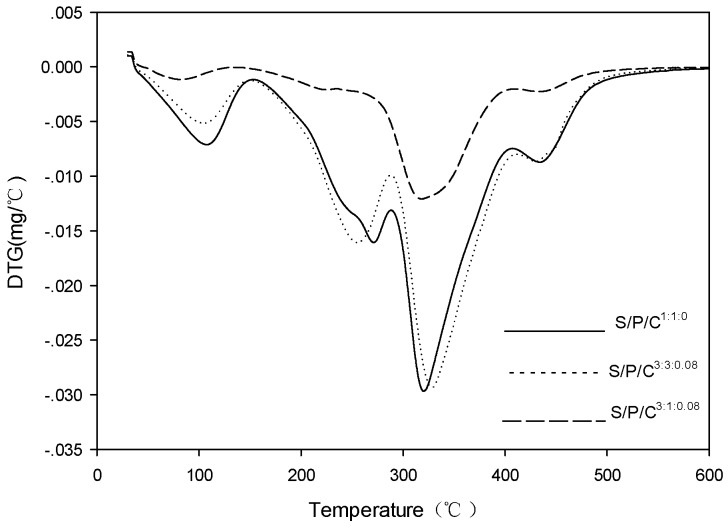
DTG thermograms of starch/polyvinyl alcohol/citric acid ternary blend functional food packaging films.

The films were tested for their thermal stability using a thermogravimetric analyzer (TGA), and the resulting TGA curves are shown in Figure 3. The derivative thermogravimetric analysis (DTG) curves are shown in Figure 4. The thermo-gravimetric curves show that the films with decreasing weight and the DTGA curves show the maximum decomposition temperature (T max) of thermal decomposition [40]. The films exhibited multi-step thermal decomposition.

The initial thermal decomposition of the S/P/C^1:1:0^ was observed from 90–105 °C, which was due to evaporation of water, weight loss of 2.490 mg, accounting for 10.24% of the total mass of the sample, and then the main thermal decomposition was observed in the range of 200–320 °C with the maximum decomposition rate around 310 °C, weight loss 22.395 mg, accounting for 88.118% of the total mass of the sample, which is due to starch and PVA molecules through hydrogen bonding formed by the new structure of thermal decomposition. Residuals left after the final thermal destruction at 600 °C were 52.149%, 57.025%, and 57.121% for the S/P/C^3:1;0.08^, S/P/C^3:3:0.08^, and S/P/C^1:1:0^ films, respectively. The S/P/C^3:3:0.08^ was initially observed from 90–110 °C, due to water evaporation, weight loss of 1.7589 mg, 7.387% of the total mass of the sample, and then the major thermal decomposition to the maximum decomposition rate observed in the 200–320 °C range with the maximum decomposition rate around 320 °C, weight loss 21.234 mg, accounting for 89.180% of the total mass of the sample, which is due to the modification of PVA by citric acid. This makes the starch and modified PVA molecules through hydrogen bonding form more new structure thermal decomposition [38].

Thus, the cross-linking between the modified PVA and the starch becomes more compact due to the addition of citric acid to the polyvinyl alcohol, and the stability of the films is enhanced [35].

### 3.6. Antimicrobial Activity

**Figure 5:**
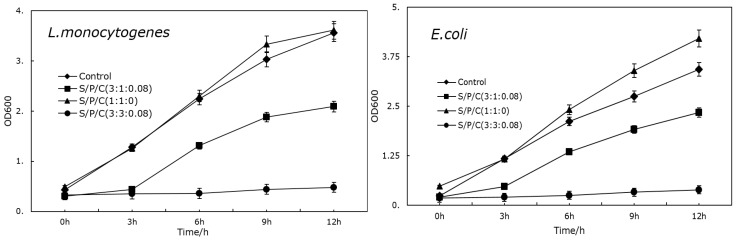
Antimicrobial activity of degradable antibacterial film against foodborne pathogenic bacteria, *L. monocytogenes* and *E. coli*.

The antibacterial activities of the ternary blends films and blank control groups against Gram-positive (*L. monocytogenes*) and Gram-negative (*E. coli*) food-borne pathogenic bacteria are shown in Figure 5. As expected, the S/P/C^1:1:0^ films did not show any antimicrobial activity against test organisms, but the concentration of bacteria compared to the blank control group was even larger while the two others with citric acid added exhibited strong antimicrobial activity against both Gram-positive (*L. mono-cytogenes*) and Gram-negative (*E. coli*) bacteria. In general, the effect of S/P/C^3:3:0.08^ was more pronounced than that of S/P/C^3:1:0.08^. It has been shown that the antibacterial mechanism of organic acid antibacterial agents is mainly to combine with the cell membrane of bacteria to break down the synthesis system between protein and cell membrane, so as to inhibit the propagation of bacteria. On the other hand, Gram-positive bacteria (*L. mono-cytogenes*) was more susceptible to the citric acid-included films than Gram-negative bacteria (*E. coli*). In addition, citric acid possesses acidity so that citric acid-added films have anti-bacterial properties, which is well-known [41].

### 3.7. Packaging Test

#### 3.7.1. Effect of Degradable Antibacterial Films on Titratable Acid (TA) Content during Storage

**Figure 6:**
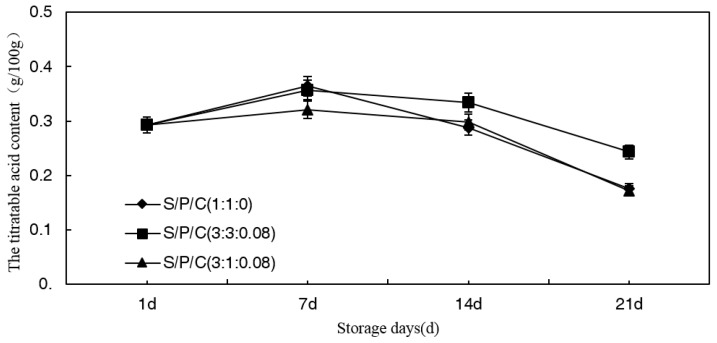
Effect of starch/polyvinyl alcohol/citric acid ternary blend functional food packaging films on the TA.

The results (Figure 6) revealed that the titratable acid (TA) content of the figs (*Ficus carica* L.) steadily increased during the first seven days of the storage. Thereafter, the titratable acid content gradually decreased. Organic acids in figs mainly include citric acid and tartaric acid, they not only can be used as respiratory matrix, which is the main source of synthetic energy ATP, but also a provider of many intermediate metabolites required for intracellular biochemical processes. As a result, the titratable acid is continuously consumed as a respiratory substrate [42,43,44]. There are researches that show there is a decrease in TA values as a natural tendency of the maturation process [45,46]. However, the titratable acid content of figs which were stored with S/P/C^3:3:0.08^ was obviously higher compared to that of the others throughout the storage period. Thus, the effect of S/P/C^3:3:0.08^ was more effective than the other types.

#### 3.7.2. Effect of Degradable Antibacterial Films on Ascorbic Acid Content during Storage

**Figure 7:**
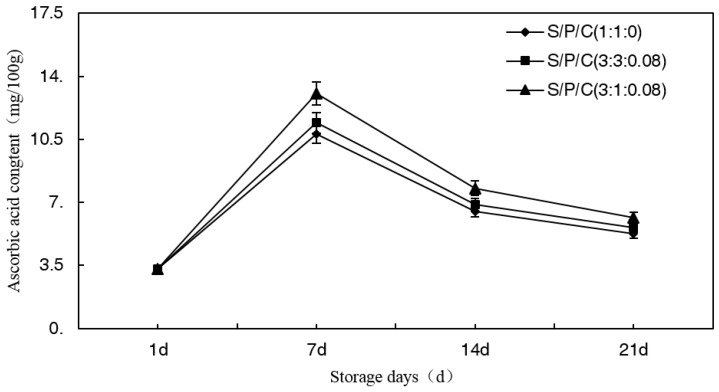
Effect of starch/polyvinyl alcohol/citric acid ternary blend functional food packaging films on ascorbic acid content during storage.

Ascorbic acid, as an antioxidant and anti-aging agent, is an essential nutrient for the human body. The ascorbic acid content can affect the fresh flavor and nutritional quality of fruits and vegetables, so it is often used as an important indicator when measuring the quality of fruits and vegetables [47]. The data presented in Figure 7 clearly show that a climacteric-like peak in the ascorbic acid content was observed in the figs on the seventh day, after that time, the ascorbic acid content gradually decreased. However, S/P/C^3:1:0.08^ had the best effect on delaying the reduction of the ascorbic acid content, followed by S/P/C^3:3:0.08^. Thus, citric acid can prevent browning as well as inhibit the decline in ascorbic acid content. Similar findings were also reported by Jiang et al. and Santerre et al. in fruits [48,49]. There were some differences in the delay of the reduction of the ascorbic acid between S/P/C^3:3:0.08^ and S/P/C^3:1:0.08^, which might be due to different levels of oxidation affected by the permeability of the films to atmospheric oxygen [50].

#### 3.7.3. Effect of Degradable Antibacterial Films on Reducing Sugar Content during Storage

**Figure 8:**
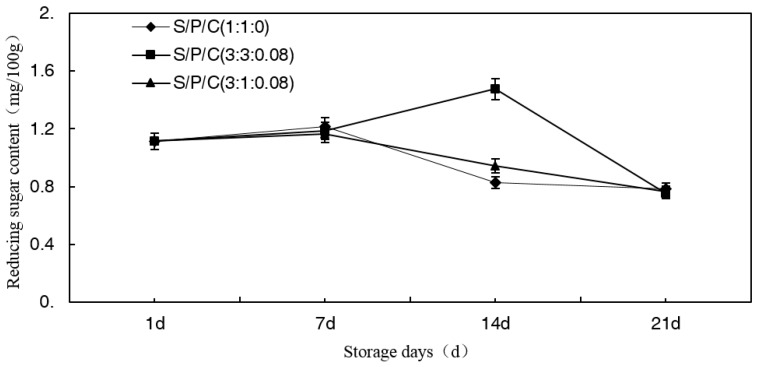
Effect of starch/polyvinyl alcohol/citric acid ternary blend functional food packaging films antibacterial and degradable film on reducing sugar.

As the time of storage increased, the reducing sugar content of the figs decreased on the whole (Figure 8). However, the content stored with S/P/C^3:3:0.08^ increased until the 14th day of the storage and then rapidly decreased. Among the treatments, S/P/C^1:1:0^ and S/P/C^3:1:0.08^ had the highest values on the seventh day. On the 14th day, S/P/C^3:3:0.08^ had the maximum value (1.476 mg/100 g). On the other hand, there were no significant differences between stored at S/P/C^3:1:0.08^ and stored at S/P/C^3:1:0.08^ with the storage time increasing. In the early stage of storage, the figs showed full ripeness whose starch was decomposed completely, and then the content of reducing sugar increased slightly. In the later period of storage, most of the reducing sugars were consumed by the respiration of the figs, which resulted in a decrease in the reducing sugar content [51,52,53]. However, the effect of S/P/C^3:3:0.08^ on the delayed reducing sugar consumption was the best.

#### 3.7.4. Effect of Degradable Antibacterial Films on Polyphenol Content during Storage

**Figure 9:**
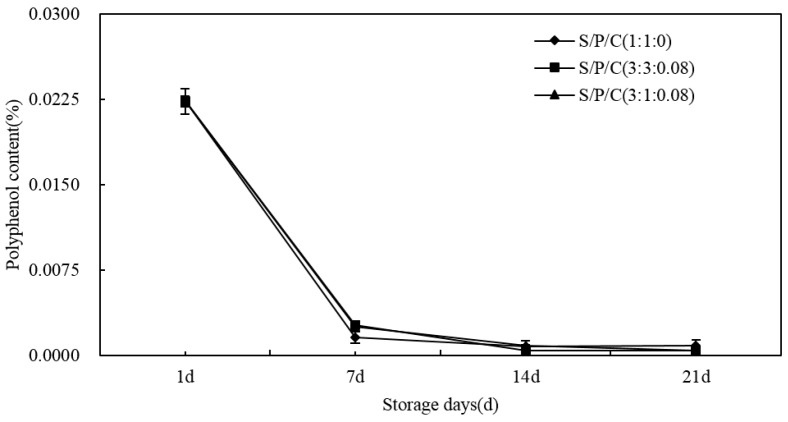
Effect of starch/polyvinyl alcohol/citric acid ternary blend functional food packaging films on the content of polyphenols.

As time of storage increased, the polyphenol content of the figs decreased overall (Figure 9). Among the treatments, the three kinds of films showed that on the first day to the seventh day levels dropped fast and then reached the lowest values on the 14th day of the storage then keeping close to zero. There were no significant differences between the films and all had a minimal effect on polyphenol. In storage, the polyphenol content fell sharply in the first week as a result of the polyphenols’ antioxidant functions which protected the cells from damage [54,55].

#### 3.7.5. Effect of Degradable Antibacterial Films on CAT Content during Storage

**Figure 10:**
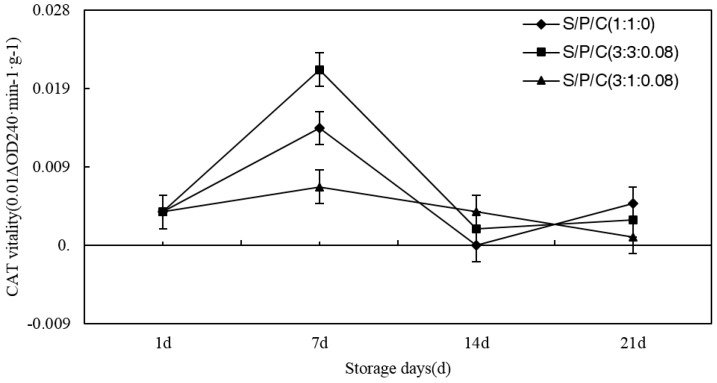
Effect of starch/polyvinyl alcohol/citric acid ternary blend functional food packaging films on the activity of CAT.

As time of storage increased, the CAT content of the figs increased after decreasing and reached the highest values on the seventh day of storage (Figure 10). CAT can act as a free radical scavenger to help remove free radicals, which play an important role in the activity of reactive oxygen species [56,57]. During the period of plants’ maturity, the CAT content of the figs increases and then during the plants’ senescence period, the CAT content of the figs decreases. The highest values of S/P/C^1:1:0^, S/P/C^3:3:0.08^ and S/P/C^3:1:0.08^ were 0.014 (0.01ΔOD240 min^–1^·g^–1^), 0.021 (0.01ΔOD240 min^–1^·g^–1^), and 0.007 (0.01ΔOD240 min^–1^·g^–1^) respectively. In conclusion, the S/P/C^3:3:0.08^ was more effective for CAT.

#### 3.7.6. Effect of Degradable Antibacterial Films on MDA Content during Storage

**Figure 11:**
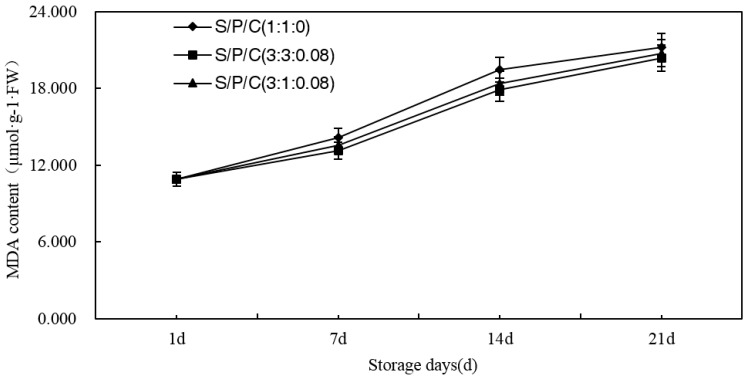
Effect of starch/polyvinyl alcohol/citric acid ternary blend functional food packaging films on the content of MDA.

As time of storage increased, the reducing sugar content of the figs increased on the whole. The results revealed that the MDA content of the figs steadily increased during the storage (Figure 11). However, in comparison, the S/P/C^1:1:0^ had the fastest growth, the S/P/C^3:1:0.08^ increased slowly, and the S/P/C^3:3:0.08^ was the lowest. During the process of storage, MDA was used as a standard that assess the degree of oxidation and the capacity to resist environmental change [58]. Excessive MDA can cause oxidative deterioration of fresh figs. Citric acid is an antioxidant synergist that enhances the antioxidant resistance of the antioxidant, thereby reducing the reduction of MDA and the permeability of the membrane to maintain the freshness of the fig.

## 4. Conclusions

Various composite films were prepared using different amounts of citric acid, different drying times, and different proportions of major components. When the concentration of citric acid in the mixed starch/PVA/citric acid composite film was 1.0%, the calcination time was about 270 min, and the tensile strength of the membrane was significant. In addition, the results of the fresh fig-packed test showed that citric acid-doped ternary blend film acids effectively prevented fruit from corrupting as well as preventing fogging on the surface due to its water vapor permeation function, and also proved ternary. The blend films have high water holding capacity and high water resistance. The S/P/C^1:1:0^ film did not show any antimicrobial activity against test organisms, but the concentration of bacteria compared to the blank control group was even larger while the two others with citric acid added exhibited strong antimicrobial activity against both Gram-positive (*L. mono-cytogenes*) and Gram-negative (*E. coli*) bacteria. In summary, the S/P/C^3:1:0.08^ and S/P/C^3:3:0.08^ composites have high potential for packaging highly breathable fresh agricultural products as antifogging packaging films and active food packaging systems due to their strong antibacterial (*E. coli*, *Listeria*) effect.

## Figures and Tables

**Table 1:** Test design of composition.

Film	Polyvinyl Alcohol (g)	Starch (g)	Glycerol (g)	Citric Acid (g)	Baking Time (min)
S/P/C^1:1:0^	2.81	2.81	1.87	0	120
S/P/C^3:3:0.08^	2.81	2.81	2.11	1	120
S/P/C^3:1:0.08^	3.75	1.25	2.5	1	120
S/P/C^1:1:0^	2.81	2.81	1.87	0	270
S/P/C^3:3:0.08^	2.81	2.81	2.11	1	270
S/P/C^3:1:0.08^	3.75	1.25	2.5	1	270
S/P/C^1:1:0^	2.81	2.81	1.87	0	300
S/P/C^3:3:0.08^	2.81	2.81	2.11	1	300
S/P/C^3:1:0.08^	3.75	1.25	2.5	1	300

The Starch: Polyvinyl alcohol: Citric acid (S/P/C^1:1:0^, S/P/C^3:1:0.08^ and S/P/C^3:3:0.08^) films.

**Table 2:** Test of the color differences of starch/polyvinyl alcohol/citric acid ternary blend functional food packaging films.

Film	ΔL(NBS)	Δa(NBS)	Δb(NBS)	ΔE(NBS)
Without cover	39.690 ± 0.0035	5.603 ± 0.0008	–17.503 ± 0.0002	43.740 ± 0.0028
S/P/C^1:1:0^	62.490 ± 0.0010	5.613 ± 0.0028	–17.943 ± 0.0002	65.260 ± 0.0009
S/P/C^3:3:0.08^	71.200 ± 0.0012	5.860 ± 0.0062	–18.453 ± 0.0047	73.790 ± 0.0012
S/P/C^3:1:0.08^	54.707 ± 0.0006	5.680 ± 0.0012	–17.273 ± 0.0010	57.653 ± 0.0004
S/P/C^1:1:0^	66.053 ± 0.0032	6.020 ± 0.0044	–17.890 ± 0.0011	68.687 ± 0.0031
S/P/C^3:3:0.08^	67.617 ± 0.0014	5.627 ± 0.0052	–17.963 ± 0.0002	70.190 ± 0.0013

Each value is the mean of three replicates with the standard deviation, Any two means in the same column followed by the same letter are not significantly (*p* > 0.05) different by Duncan’s multiple range tests.

**Table 3:** Tensile properties of starch/polyvinyl alcohol/citric acid ternary blend functional food packaging films.

Film	Thickness (mm)	Tensile Strength (MPa)	Elastic Modulus (%)
S/P/C^1:1:0^	0.0606 ± 0.0277	33.84 ± 1.8	27 ± 2.5
S/P/C^3:3:0.08^	0.0648 ± 0.0691	45.22 ± 2.4	66 ± 3.6
S/P/C^3:1:0.08^	0.1150 ± 0.0139	19.58 ± 1.1	27 ± 5.1
S/P/C^1:1:0^	0.0688 ± 0.0674	35.98 ± 2.5	29 ± 4.8
S/P/C^3:3:0.08^	0.1220 ± 0.0250	45.54 ± 2.6	74 ± 2.4
S/P/C^3:1:0.08^	0.1074 ± 0.0327	23.25 ± 1.5	31 ± 2.1
S/P/C^1:1:0^	0.0538 ± 0.0416	34.51 ± 2.3	27 ± 2.0
S/P/C^3:3:0.08^	0.0694 ± 0.0276	31.68 ± 2.0	36 ± 3.1
S/P/C^3:1:0.08^	0.1166 ± 0.0402	20.00 ± 2.7	21 ± 2.4

Each value is the mean of three replicates with the standard deviation, Any two means in the same column followed by the same letter are not significantly (*p* > 0.05) different by Duncan’s multiple range tests.

**Table 4:** The water vapor permeation rate and solubility of starch/polyvinyl alcohol/citric acid ternary blend functional food packaging films.

Film	WVP(×10^−9^ g·m/m^2^·Pa·s)	Water Solubility (%)
S/P/C^1:1:0^	1.15 ± 0.04	29.8 ± 0.5
S/P/C^3:3:0.08^	1.21 ± 0.05	41.1 ± 0.2
S/P/C^3:1:0.08^	0.36 ± 0.11	6.5 ± 0.12
S/P/C^1:1:0^	1.56 ± 0.09	36.1 ± 0.04
S/P/C^3:3:0.08^	1.95 ± 0.15	45.6 ± 0.3
S/P/C^3:1:0.08^	0.42 ± 0.08	7.0 ± 0.7
S/P/C^1:1:0^	1.64 ± 0.10	33.5 ± 0.2
S/P/C^3:3:0.08^	1.76 ± 0.03	40.9 ± 0.14
S/P/C^3:1:0.08^	0.21 ± 0.13	6.2 ± 0.6
